# Violence against women: The perspective of academic women

**DOI:** 10.1186/1471-2458-10-490

**Published:** 2010-08-17

**Authors:** Sibel Kalaca, Pınar Dundar

**Affiliations:** 1 Marmara University School of Medicine, Dept. of Public Health, Istanbul; 2 Celal Bayar University School of Medicine, Dept. of Public Health, Manisa

## Abstract

**Background:**

Opinion surveys about potential causes of violence against women (VAW) are uncommon. This study explores academic women's opinions about VAW and the ways of reducing violence.

**Methods:**

Quantitative and qualitative methods were used in this descriptive study. One hundred-and-fifteen academicians participated in the study from two universities. A questionnaire was used regarding the definition and the causes of VAW, the risk groups and opinions about the solutions. Additionally, two authors interviewed 8 academicians from universities other than that of the interviewing author.

**Results:**

Academicians discussed the problem from the perspective of "gender-based violence" rather than "family violence". The majority of the participants stated that nonworking women of low socioeconomic status are most at risk for VAW. They indicated that psychological violence is more prevalent against educated women, whilst physical violence is more likely to occur against uneducated and nonworking women. Perpetrator related factors were the most frequently stated causes of VAW. Thirty-five percent of the academicians defined themselves as at risk of some act of VAW. Recommendations for actions against violence were empowerment of women, increasing the educational levels in the society, and legal measures.

**Conclusions:**

Academic women introduced an ecological approach for the explanation of VAW by stressing the importance of taking into account the global context of the occurrence of VAW. Similar studies with various community members -including men- will help to define targeted interventions.

## Background

For over three decades, women's advocacy groups around the world have been working to draw more attention to the physical, psychological, and sexual abuse of women and to stimulate action [1]. The movement in Turkey is a part of this advocacy. When feminists in Turkey first used the term "domestic violence" in 1987, they were treated as a group of marginal women [2]. Since then important steps have been taken in Turkey in the struggle to eliminate violence against women (VAW). In recent years, gender-based violence has occupied the national agenda, particularly in the context of reforms in basic laws such as the Civil Code and the Turkish Penal Code (TCK) [2]. Today, with recent reforms of these laws relating to women's rights, Turkey comes close to meeting EU standards. However, the problem is not easy to solve by legislation alone. The lives of many women in Turkey are still shaped by social and cultural forces, rather than by constitutional rights and freedoms.

According to the United Nations definition, the term VAW means any act of gender-based violence that results in, or is likely to result in, physical, sexual or psychological harm or suffering to women, including threats of such acts, coercion or arbitrary deprivation of liberty, whether occurring in public or in private life [1]. Increasingly, research has focused on the inter-relatedness of various factors that can improve our understanding of the problem within different cultural contexts [3]. More and more researchers are using an "ecological framework" to understand the interplay of personal, situational and socio-cultural factors that combine to cause violence. As a brief definition, "an ecological approach to abuse conceptualizes violence as a multifaceted phenomenon grounded in an interplay among personal, situational, and socio-cultural factors" [4]. In this framework VAW results from the interactions of factors at different levels of the social environment such as individual levels, the level of the family and relationship at the community level or at the societal level [1].

In Turkey, there have been several nationwide studies which provide data about the situation on VAW in Turkey. According to the National Research on Domestic Violence Against Women in Turkey (2008) [5] violence by husbands is the most common form of violence in women's lives. The study also revealed that there is considerable variation between regions. Between 26% and 57% of ever-married women experienced physical or sexual violence by a husband in their lives. According to the results of another nationwide study which was done in 1993-94 [6], the percentage of women reporting physical violence in the family was 35% whereas it was 53% for psychological violence. Based on its qualitative data, this study concluded that "the majority of women who have been subjected to violence have a tendency to 'normalize' violence as a concept". In another recent nationwide study which was done in 2007, it was found that that nine out of ten women agreed with the statement that "wife-beating" was never justifiable and, nine out of ten women said "yes" to the question of whether the courts should "penalize" the men who exercise violence against their wives [2].

Population-based perception studies on potential causes of VAW may enlighten context-relevant primary preventive actions in settings where data are limited [7]. The present exploratory study was performed on a group of women who teach at the university. In the study, we did not ask about their experiences of specific acts of violence rather aimed to evaluate their views, explanations about already documented potential causes of VAW and the ways of prevention. Although any woman can be the victims of violence, there are some factors that may protect some women from VAW. For example, women who have authority and power outside the family -such as academic women- tend to experience lower levels of abuse in an intimate partnership [1]. On the other hand, there is evidence that women's socio-demographic characteristics and prior victimization did not much affect their opinions about causes of VAW [7].

This study, conducted in two universities, deals with how academic women explain VAW, how they relate different levels of risk factors, and what prevention strategies they propose.

## Methods

This descriptive study was done in the Haydarpasa Campus of Marmara University (HMU) in Istanbul and in Celal Bayar University (CBU) in Manisa. One hundred and fifteen academic women participated in the study from different faculties of these universities. Quantitative and qualitative methods were used. We followed the guide of the World Health Organization (WHO) when developing the research strategy; using the term "violence against women" (VAW) to refer to the full range of abuses [1].

In the quantitative part of the study, a structured questionnaire was used. The greater part of the questionnaire consisted of closed-ended questions. There were also open-ended questions to elaborate responses to the closed questions and to allow the respondents to identify new issues not captured in the closed questions [1,8]. All five faculties in HMU (Medical Faculty, Faculty of Pharmacy, Faculty of Law, School of Nursing and Faculty of Fine Arts) were included in the study. In CBU, academics from the Medical Faculty, School of Nursing, Vocational School of Allied Health Professions, and School of Physical Education participated in the study. All departments in each faculty were visited and academic women who hold assistant, associated or full professor position were given the questionnaire. Each academic was visited at least three times when they were out of office. Formal ethical approval was not sought as there was no requirement for this study, due to the observational nature of the research. Academics were explicitly informed of the aims, methods and the practical implications of the study and their participation was voluntary. Questionnaires were distributed in closed envelopes and participants were asked to fill the questionnaire anonymously. There was one refusal. Two or three days later, researchers gathered the questionnaire using a voting box. All results were anonymous and the confidentiality of the subjects was protected.

At the end of the questionnaire, women were asked whether they were willing to participate in an in-depth interview about VAW; and if so, were asked to provide their contact information. Fifty academics (43.9%) agreed to participate in the interview. From these we have chosen 8 women. For the selection, first we excluded subjects who did not give their contact information. Secondly we considered the academic faculties of the candidates in order to have the different faculties represented. When there was more than one volunteer from one faculty we considered the answers to questionnaire in order to have a diversity of given answers. Two researchers did 8 in-depth interviews (4 each) with the academics, so that the researcher and the academic were not from the same University. The main reasons of having a qualitative component in this study was to get a better understanding about the views of the academicians; besides, we wanted to elaborate responses to open questions in the questionnaire such as *"chose five words to describe violence against women". *During the interview, interviewees were given their questionnaire and their clarifications for some particular topics were requested.

Appointments were fixed and interviews were done by one researcher in the interviewee's office. Each interview took approximately 40-45 minutes. Three of the eight interviews were not tape recorded since interviewees did not give permission. In this case, the interviewer took notes. For the interview a semi-structured topic guide was used [9]; although many questions arose naturally during the interview.

The SPSS 13.0 program was used for quantitative data entry and analysis. For the interview, responses were classified into sub-themes that were independently assigned by two researchers; discrepancies were reviewed and jointly agreed.

## Results

Means ± standard deviations of age, professional life-span, and academic life-span were as follows (in years): 40.3 ± 7.9, 16.9 ± 7.6, 12.0 ± 7.9. Of the participants, 47% were from the medical faculty, followed by the school of nursing (24.3%) and the faculty of pharmacy (11.3%).

The academic women were asked to provide 5 words to describe VAW. The most frequently used words were "*humiliation-insulting*", "*beating*", "*primitiveness/wildness/inhuman*", and "*inferiority complex/incompetence/lack of confidence/unsatisfied ego*". Based on an open-ended question, women reported that non-working, uneducated and poor women, women who have no economic freedom and no social security are most at risk for VAW (Table 1). The response to the same question also included a completely different group of women: well educated, working and self confident women were also acknowledged as risk groups for VAW.

**Table 1:** Women at risk for VAW (Manisa-İstanbul, 2008)

Women at risk for VAW	n	%
Non-working, women who have no economic freedom and no social security	52	22.1
Uneducated, low educational attainment	47	20.0
From low socioeconomic status, poor women	23	10.0
All women	17	7.2
Well educated women	17	7.2
Women who lack self confidence, passive, dim	12	5.1
Rural women, Eastern origin women	11	4.6
Working women, women with economic freedom	10	4.2
Women from a traditional community, women from a repressive community, women from an extended family	8	3.4
Prostitutes	7	2.9
Other	31	13.3

Total*	235	100.0

* Number of answers

According to 59% of the academics (68 women), violence has varied according to the status of the women. Psychological and verbal violence are more prevalent against educated women, whereas physical violence is more likely to occur against uneducated and nonworking women. Ten women (8.7%) indicated that educated women were less likely to experience any type of violence. Their explanation for this phenomenon was indicated by their image of the educated women's husbands, who were thought likely to be educated too. Interestingly, two women stated that "when women have a higher status, they can themselves be the perpetrators of violence". Data was not shown.

Perpetrator related factors were at the top of the list of causes of VAW. Women mostly focused on the individual aggressor: alcohol or drug use was also included in most of the answers. The second leading cause indicated gender related factors such as gender roles, the patriarchal nature of society, the culture, and norms granting men control over female behavior (Table 2). Lack of education was reported as another important cause of VAW. When we analyzed the statements, we saw that "lack of education" was not attributed to a certain sex; rather it has been used as a general term indicating that a low educational level in a society is related to VAW.

**Table 2:** Causes of violence against women (Manisa-İstanbul, 2008)

Causes	n	%
Perpetrator related factors:	79	34.8
▪ *Lack of self-confidence, feeling of insignificance-inadequacy, inferiority complex, ego satisfaction*	*54/79*	*68.3*
▪ *Mental health problems, alcohol or drug abuse*	*25/79*	*31.7*
Gender or gender inequity based causes	52	22.9
Economic problems	50	22.0
Lack of education	34	15.0
Witnessing family violence as a child	12	5.3

Total*	227	100.0

* Number of answers

We asked whether in some situations academics would consider violence justified. There was one "yes", and one "may be" answer; both respondents refused to participate in the in-depth interview. Academics were given 6 items and asked to indicate for each if it was related to the perpetration of VAW or not. Intimate partners were marked by 91.2% of the participants. (Table 3). In total women mostly referred "gender-based violence" by indicating men to be the main perpetrators of violence.

**Table 3:** Perpetrators of violence (Manisa-İstanbul, 2008)

Perpetrators	n (yes)	%*
Current or former intimate partner, spouse, lover	103	91.2
Any male relative	73	64.6
Community	47	41.6
Female managers	35	31.0
Male managers	33	29.2
Women from close environment or from family	31	27.4

*percentages are based on total number of women (113) who answered the question

We asked three questions specific to academic women. Almost 70% of the academics (78 women) stated that academic women were at risk of VAW. Half of them knew other academics in their close environment who have been experiencing domestic violence, and 35% of them stated that they see themselves at risk of some act of VAW. Academics who indicated themselves as at risk, were asked to provide a short explanation regarding the sources/causes and/or means of violence. The main focuses of the explanations were as follows: *blocking academic progress, emotional-verbal violence at home or at work, mobbing, violence outside home *(in traffic, shopping, in whole life).

Empowerment of women was the most cited strategy for prevention of VAW based on an open ended question. Increasing the educational levels of the whole society was indicated as the second strategy (Table 4).

**Table 4:** Recommendations for action (Manisa-Istanbul, 2008)

	n	%
Empowerment of women	100	33.2
Education	48	15.9
Legislation and legal system	36	12.0
Education of boys at home, role modeling	30	10.0
Support groups, NGOs, shelters	26	8.6
Raising awareness in the community	25	8.3
Media support	16	5.3
Education of men	11	3.6
Making no disguises, uncovering violence when it happen	9	3.0

Total*	301	100.0

* number of answers

### In-depth interview

Eight in-depth interviews were held with academics from different faculties. Generally, interviewees expressed the view that the frequency of VAW was not so different in Turkey compared to other countries. Almost all of them emphasized that the eastern part of Turkey was different than the western part of Turkey as regards the nature; the social acceptability of VAW and the circumstances in which it occurs. Their perception was that women from the East were the most oppressed; VAW was more acceptable to the society and honor killing was one of the most important problems. Although there are striking differences to the disadvantage of women living in Eastern regions of Turkey regarding education, income and health indicators, no significant differences were found between East and West in terms of experiences of violence or their views on the struggle against violence [2].

Almost all of the interviewees suggested gender-based power relationships and inequitable gender norms for consideration. The power relationship between men and women was introduced as the main point of conflict regardless of the status of women.

" women are perceived as servants.. this is the same in the west and among educated women. She works at the/her workplace, but she is expected to serve at home too... this role is given by her family ... it will take time to change.."

Traditional and rigid views of gender roles, ideas of men being dominant and controlling were presented as the facts behind the "male violence". In addition, impotence and desperation were referred as explanations of the behavior of the perpetrator by most of the interviewees.

"...because of impotence and desperation... in fact, the perpetrator is the one in a fix. man tries to cover his own impotence/weakness up... tries to cover it up by proceeding to violence towards those who have less power than he has'.. it is the same between mother and child. I mean, big fish swallow small fish.."

As a common opinion, all participants claimed that for women, "having power" protects them against violence, while it is also a risk factor itself.

"powerful women stimulate the violence; at the same time, power is a deterrent..."

Gender based power relationships were also introduced in the academic setting. One interviewee who has been working as head of a department complained about her women colleagues, who did not want to accept her leadership.

*"in the academic world it is difficult for women to be in a high position....mostly other women are reacting to this; they cannot accept it, they are jealous of her.. Because the "normal" is that the man gets the power and is the manager... *so, *she asks herself what superiority has she got... but *the *men already have got the superiority.. "*

Generally participants stated that there was no gender discrimination in the academic world regarding academic promotion since all criteria necessary for academic progress were well established and standardized.

In the in-depth interview when we came to prevention, "education" was one of the main tools for fighting against VAW. Education of both men and women, as well as training for good parenting practices were included in the "education" strategy. One academician especially emphasized three points: education of teachers, solidarity at schools instead of competition, and co-education.

Even though the participants indicated that "powerful women" may be a risk factor themselves, they strongly emphasized women's empowerment as one of the important preventive strategies for combating VAW. Three participants recommended that all types of violence be considered, in all their aspects and of all types. For example, violence against children, violence against the elderly, violence at school, violence in the media. The media was given an important role in opposing violence with particular attention to discriminatory programs that reinforce existing gender roles. One participant indicated the necessity for men to participate in opposing VAW. A further issue was about responding to violence both at the individual and the community level. All participants strongly argued that abused women should be brave, should not hide, should not flee and should report their victimization even when they fear the social stigma. Finally, penalizing the perpetrator was the most emphasized issue regarding legislative measures.

## Discussion and Conclusions

In this study we have evaluated academic women's opinions on the definition and causes of violence and the ways opposing the violence. Although the response rate was quite high, this is still not a representative sample of academics in the two universities; since only two campuses of those universities were selected based on the convenience of the sampling approach. Therefore, the degree to which the sample differs from the population remains unknown. However, there are no data in the literature about how women having a high status understand, explain and discuss this important public health problem. In the study, academic women were asked to provide 5 words to describe VAW. Mainly, three groups of words were provided; words referring to types of violence such as "beating" and "humiliating"; words relating to the perpetrators such as "*inferiority complex/unsatisfied ego" *and words referring to their response to VAW: *primitiveness/wildness/inhuman*. None of them used the terms "domestic violence" or "family violence". Many of the most commonly used terms for referring VAW have different meanings in different regions, and are derived from diverse theoretical perspectives and disciplines [1]. Although "family violence" refers to all forms of abuse within the family regardless of the age or sex of the victim or the perpetrator; it does not encompass the many types of violence to which women are exposed outside the home. There is an increasing international consensus that the abuse of women and girls, regardless of where it occurs, should be considered as "gender-based violence", as it largely stems from women's subordinate status to men in society [1]. In the present study we have seen that academic women discussed the problem from the perspective of "gender-based violence" rather than "family violence". Under the "violence against women" concept, they mentioned violence in the street and violence at the workplace, as well as violence in the home. In addition, academic women introduced an ecological approach to the explanation of gender-based violence, by indicating VAW as resulting from the interaction of factors at different levels of the social environment [1,10]. On the other hand, academics were aware of the fact that one of the most common forms of violence against women was that perpetrated by a husband or other intimate male partner [1,11].

It is not easy to compare our results to those of other studies, as opinion surveys about potential causes of VAW are uncommon. In their study from Iran, Hamzeh et al. found that, social and cultural factors were not mentioned as much as potential other factors by married women; for whom substance abuse and men's mental problems ranked high [7]. Their findings were consistent with another study from the USA, which was a public opinion survey on domestic violence [12]. In the present study, perpetrator related factors were also reported as the leading factors behind the VAW. Women mostly focused on the reasons for the perpetrator's behavior -mostly related to the intimate nature of the partnership- such as lack of self-confidence, feeling of insignificance or inadequacy. In the qualitative part of the study, the interviewees were asked for clarifications of some particular topics. Based on these results, we found that women associated behaviors of the perpetrators with gender roles which push men to be strong, brave and successful.

Violence against women is not only a manifestation of sex inequality, but also serves to maintain this unequal balance of power. In some cases, perpetrators consciously use violence as a mechanism for subordination. For example, violence by intimate partners is often used to demonstrate and enforce a man's position as head of the household or relationship [11]. Likewise, in the qualitative part of the study, almost all of the interviewees indicated that the power relationship between men and women was the main point of conflict regardless of the women's status. Participants claimed that women who earn more money than their husbands are at increased risk of physical violence. This has also been found in some other countries; that is women's increasing economic activity and independence is viewed as a threat which leads to increased violence. This is particularly true when the male partner is unemployed, and feels his power undermined in the household [3]. This was also one of the key findings of a VAW study from Turkey [2] which showed that women with higher incomes than their spouses are at double the risk of a beating.

Alcohol or drug uses were also included in most of the answers as in previous studies [7,12]. Economic problems and childhood experience of violence have also been mentioned as important risk factors in the perpetrator. Experiences during childhood such as witnessing domestic violence and experiencing physical and sexual abuse have been identified as factors that put children at risk [3,11,13].

In the present study, the majority of the participants stated that nonworking women from a low socioeconomic status are most at risk for VAW. In a recent review of nationally representative surveys in nine countries it was found that for women, low educational attainment, being under 25 years of age, having witnessed her father's violence against her mother, living in an urban area, and low socio-economic status were consistently associated with an increased risk of abuse [14]. According to a WHO Multi-Country Study [10], higher education was associated with less violence in many settings. In another study it was concluded that higher socioeconomic status and good social support acted as protective buffers against spousal physical violence [15]. According to the National Research on Domestic Violence Against Women in Turkey [5], violence has been decreasing as the educational level of women has increased. As the academic women in the present study indicated, there is a meaningful statistical relationship between both the woman's and her husband's educational status and the rate of physical violence, and this has also been found in another recent nationwide study in Turkey [2]. While half of the women whose husbands are illiterate say they have been physically abused at least once, the figure for those whose husbands have a college/university education is 18%. The authors concluded that these data must be interpreted cautiously because the higher the woman's educational level and socioeconomic status, the more difficult it becomes for her to admit to having experienced violence [2].

In the present study, only two academic women out of 115 (1.7%) said that under certain circumstances violence could be justified. Although this is a low percentage, it is still a remarkable finding to see that there are women among a group of academics who legitimize VAW in some situations. Women expressed that there was no gender discrimination regarding academic promotion. A similar finding was also reported in another study conducted with senior academic managers in Turkey and Portugal [16]. Although university regulations do not have a rule that prevents academic women from being academic managers, women may not be willing to take administrative responsibility due to the traditional social roles of women. Furthermore, when we looked at given examples and some experiences in this study, we found that the academic world was not free of existing gender roles. This may indicate unnoticed gender discrimination in the academic world.

Women's empowerment was suggested as one of the most important prevention strategies together with increasing the educational levels of society. Although legislative arrangements were strongly recommended, none of academic women mentioned the recent reforms of laws relating to women's rights. However, this was not asked specifically.

In the present study we found that women's socio-demographic characteristics did not much affect their opinions about causes of VAW. However, academic women introduced an ecological approach for the explanation of VAW. In order to get a better understanding of the gendered power relations behind VAW, similar studies with various community members -including men- are needed. Gender discrimination at universities should be further investigated as an important research topic.

## Competing interests

The authors declare that they have no competing interests.

## Authors' contributions

SK conceived of the study, developed the questionnaire, conducted the interviews, performed the statistical analysis and finalized the manuscript. PD participated in the study design, developed the semi-structural interview guide, conducted the interviews, and helped to draft the manuscript. All authors read and approved the final manuscript.

## Pre-publication history

The pre-publication history for this paper can be accessed here:

http://www.biomedcentral.com/1471-2458/10/490/prepub
